# Bilateral Conjunctival Small Lymphocytic Lymphoma Simulating Inflammatory Ocular Surface Disease: A Case Report

**DOI:** 10.3390/vision10040068

**Published:** 2026-09-08

**Authors:** Maria Vivas, Júlio Almeida, Catarina Monteiro, Mara Ferreira, Isabel Prieto

**Affiliations:** Hospital Prof. Doutor Fernando Fonseca, 2720 Lisboa, Portugal

**Keywords:** conjunctival lymphoma, small lymphocytic lymphoma, ocular adnexal lymphoma, Bruton tyrosine kinase inhibitor, salmon patch, ibrutinib

## Abstract

Conjunctival lymphoma classically appears as a painless salmon-pink subepithelial infiltrate, but its indolent forms can closely imitate inflammatory ocular surface disease and delay diagnosis, particularly when an uncommon subtype such as small lymphocytic lymphoma presents bilaterally and in isolation. A woman in her early fifties presented with one month of left ocular discomfort and sectoral redness, and slit-lamp examination revealed two multilobulated hyperaemic bulbar conjunctival nodules, clinically indistinguishable from nodular episcleritis. Topical corticosteroids and a topical non-steroidal anti-inflammatory drug relieved the ocular discomfort and hyperaemia, but the nodules regressed only partially and at no point resolved. This dissociation between symptomatic relief and persistence of the lesions was, in retrospect, the earliest argument against a purely inflammatory process. The initial work-up pointed toward inflammatory and granulomatous causes: elevated serum angiotensin-converting enzyme and lysozyme raised the possibility of sarcoidosis, although thoracic computed tomography, bronchoscopy and a first conjunctival biopsy performed without a lymphoma-directed panel were unrevealing, showing only reactive lymphoid hyperplasia. Approximately one year later, recurrent and now bilateral disease prompted a repeat biopsy with directed immunohistochemistry, which demonstrated a CD20-positive small B-cell infiltrate co-expressing CD5 and CD23 with negative cyclin D1 and CD10, consistent with chronic lymphocytic leukaemia/small lymphocytic lymphoma. Systemic staging with ^18^F-FDG PET/CT and bone marrow biopsy revealed no definite extra-conjunctival disease. After multidisciplinary review of observation, local radiotherapy and surgical excision, systemic therapy was preferred given the bilateral, recurrent and symptomatic course, and oral ibrutinib 420 mg once daily achieved complete clinical regression at three months and a sustained ocular response at two years, with indefinite haematological and ophthalmological surveillance planned.

## 1. Introduction

Ocular adnexal lymphoma is an uncommon malignancy that arises in the orbit, conjunctiva, lacrimal gland or eyelids and is, in the great majority of cases, a low-grade B-cell neoplasm. Conjunctival involvement characteristically manifests as a painless salmon-pink subepithelial infiltrate of the bulbar conjunctiva or fornices, which may be unilateral or bilateral, and approximately 80% of cases correspond to extranodal marginal-zone lymphoma of mucosa-associated lymphoid tissue [1]. The other subtypes are far less common: chronic lymphocytic leukaemia/small lymphocytic lymphoma (CLL/SLL) accounts for only about 5% of ocular adnexal lymphomas [2], and, although ophthalmic disease may develop during the course of systemic CLL, the conjunctiva is among its least frequently involved sites [3]. Primary conjunctival CLL/SLL presenting as the initial and isolated manifestation of disease, without a known haematological diagnosis, is rarely reported, and the few documented cases have been predominantly unilateral [4,5].

The principal difficulty in recognising conjunctival lymphoma lies in its resemblance to benign inflammatory disease. Indolent nodular lesions are clinically indistinguishable from far more common entities such as nodular episcleritis, follicular conjunctivitis, sarcoidosis, IgG4-related disease and reactive lymphoid hyperplasia [1]. This overlap predisposes to diagnostic anchoring, whereby a lesion that persists or recurs may be attributed to the presumed inflammatory process rather than prompting reconsideration of the diagnosis, and definitive management may be delayed by months or years. Such delay carries prognostic weight, as conjunctival lymphoid tumours that appear localised at presentation progress to systemic lymphoma in 7%, 15% and 28% of patients at one, five and ten years respectively, with a higher risk in bilateral disease [6]. We report a case of bilateral conjunctival CLL/SLL that was managed as inflammatory ocular surface disease for approximately one year, through a non-diagnostic biopsy and a sarcoidosis work-up, until recurrence raised the suspicion of a neoplastic process and prompted a repeat biopsy with comprehensive immunophenotyping, which established the diagnosis. Conjunctival CLL/SLL can therefore closely mimic inflammatory ocular surface disease, and its distinction rests on lymphoma-directed immunophenotyping rather than on morphology alone.

## 2. Detailed Case Description

**Figure 1:**
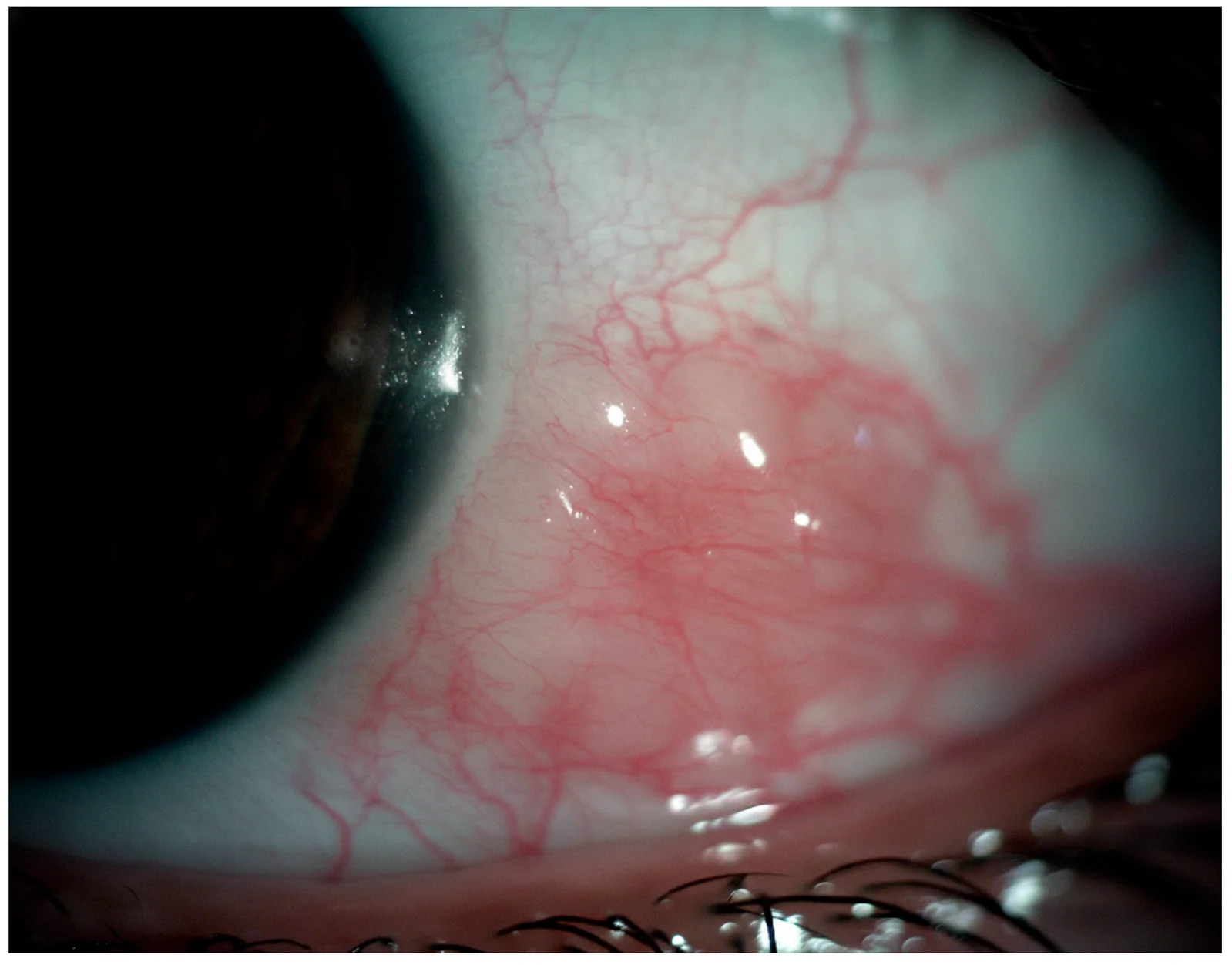
Slit-lamp photograph of the left eye at presentation, showing a multilobulated hyperaemic nodule of the bulbar conjunctiva, clinically indistinguishable from nodular episcleritis.

A 51-year-old woman with no relevant past medical history presented with a one-month history of left ocular discomfort and sectoral redness, without visual loss. Best-corrected visual acuity was 20/20 in each eye. Slit-lamp examination of the left eye revealed two multilobulated hyperaemic nodular lesions on the temporal bulbar conjunctiva (Figure 1). The anterior chamber was quiet, without cells or keratic precipitates, the intraocular pressure was normal, and the fundus was unremarkable. The right eye was normal.

The lesions were interpreted as inflammatory and treated with topical corticosteroids three times daily and a topical non-steroidal anti-inflammatory drug, while an aetiological work-up was undertaken in parallel. The ocular discomfort and hyperaemia improved on this regimen, but the nodules themselves regressed only partially and at no point disappeared. This dissociation between a symptomatic response and the persistence of the lesions was, in retrospect, the first argument against a self-limited inflammatory process; at the time, it was attributed to incomplete control of a presumed nodular episcleritis. Serum angiotensin-converting enzyme and lysozyme were elevated, raising the possibility of sarcoidosis, whereas the complete blood count and the remaining investigations were unremarkable. This prompted thoracic computed tomography, bronchoscopy and bronchoalveolar lavage, none of which showed granulomas or neoplastic infiltration.

Approximately two months after presentation, the nodules had become less prominent but had not resolved, and an excisional biopsy of the left temporal lesions was performed. Histopathology showed the conjunctival substantia propria expanded by a lymphoid infiltrate containing a reactive-appearing lymphoid follicle with a germinal centre, set in a background of non-specific chronic inflammation, without granulomas, necrosis or cytological atypia. Because the clinical and laboratory context pointed towards granulomatous disease, the specimen was examined with the panel appropriate to that question, and no lymphoma-directed immunophenotyping, assessment of κ/λ light-chain restriction or molecular clonality study was requested. The report was issued as reactive lymphoid hyperplasia. In keeping with this benign result, topical anti-inflammatory treatment was continued under close clinical surveillance.

**Figure 2:**
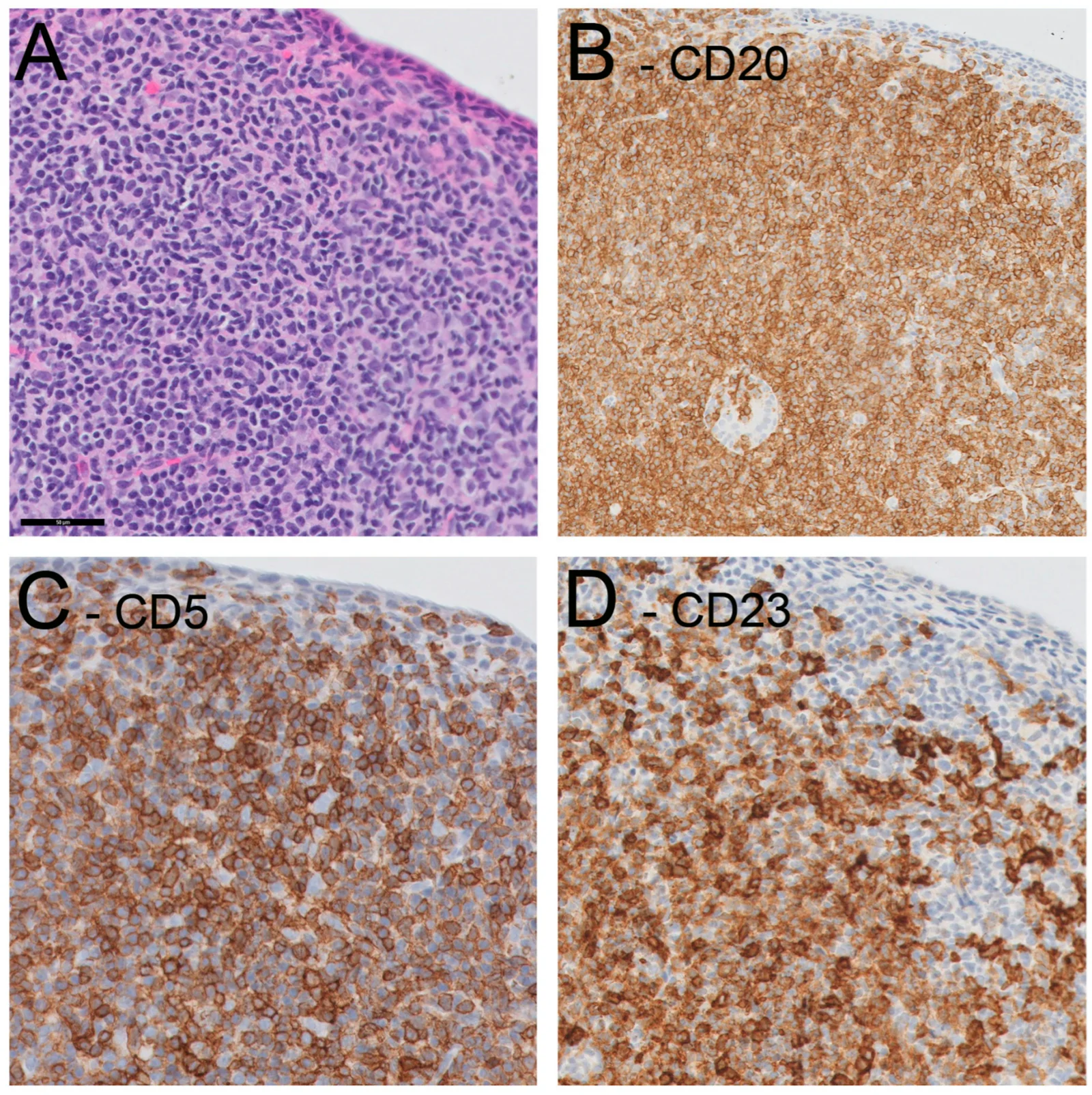
Histopathology and immunohistochemistry of the conjunctival biopsy. (**A**) Haematoxylin and eosin staining shows a dense subepithelial infiltrate of small mature lymphocytes without germinal-centre formation. The infiltrate is diffusely and strongly positive for CD20 (**B**) and co-expresses CD5 (**C**) and CD23 (**D**), consistent with chronic lymphocytic leukaemia/small lymphocytic lymphoma. Scale bar, 50 µm (**A**).

Over the following months the lesions persisted despite anti-inflammatory treatment, with new nodules of similar morphology in the nasal bulbar conjunctiva of the left eye and in the right eye, establishing bilateral multifocal conjunctival involvement. Four features together displaced the inflammatory hypothesis: a course far more protracted than that of nodular episcleritis, which is typically self-limited; the absence of true resolution despite topical corticosteroid; recurrence at a site that had already been excised; and progression from unilateral to bilateral, multifocal disease. Bilaterality was the most decisive, being distinctly uncommon in nodular episcleritis, yet well recognised in ocular adnexal lymphoma, in which bilateral involvement is reported in approximately 7–24% of cases [5]. A repeat excisional biopsy was performed and submitted with an explicit request for a comprehensive lymphoma-directed immunohistochemical panel. Histopathology demonstrated a dense subepithelial infiltrate of small mature lymphocytes with condensed chromatin and scant cytoplasm, without germinal-centre formation (Figure 2A). On immunohistochemistry, the infiltrate was diffusely and strongly positive for CD20 (Figure 2B) and co-expressed CD5 (Figure 2C) and CD23 (Figure 2D), while cyclin D1 and CD10 were negative.

This small mature B-cell phenotype, CD20-positive, CD5-positive and CD23-positive with negative cyclin D1 and CD10, was diagnostic of chronic lymphocytic leukaemia/small lymphocytic lymphoma. The co-expression of CD23 with absent cyclin D1 argued against mantle cell lymphoma, which is characteristically CD23-negative and cyclin D1-positive, and the absence of CD10 argued against follicular lymphoma. The diagnosis was confirmed on specialist haematopathology review.

The patient was referred to haematology–oncology to determine whether the conjunctival disease was isolated or a manifestation of systemic CLL/SLL. Because of the extranodal location, whole-body ^18^F-FDG PET/CT was performed in preference to conventional computed tomography. It showed bilateral submandibular and laterocervical lymph nodes of low-to-moderate metabolic activity and indeterminate significance, together with diffuse osteomedullary hypermetabolism, whereas the bone marrow biopsy with flow cytometry showed no infiltration, and there was no peripheral blood lymphocytosis or cytopenia. No TP53 mutation was identified. In the absence of definite nodal, marrow or peripheral-blood involvement and of B symptoms and with a peripheral clonal B-lymphocyte count below 5 × 10^9^/L, the disease was classified as isolated bilateral conjunctival CLL/SLL, in accordance with the International Workshop on Chronic Lymphocytic Leukaemia (iwCLL) definition of SLL [7]. The term isolated is used here to denote disease confined to the conjunctiva by current staging criteria and not to imply that occult systemic disease was excluded. The cervical nodes were reported as of indeterminate significance rather than as definite nodal involvement and were not characterised further by biopsy, and the diffuse osteomedullary hypermetabolism, a non-specific finding, was not accompanied by marrow infiltration on bone marrow biopsy with flow cytometry. Neither finding satisfied the iwCLL criteria for nodal or marrow disease, and both were therefore kept under surveillance rather than taken as evidence of systemic involvement.

**Figure 3:**
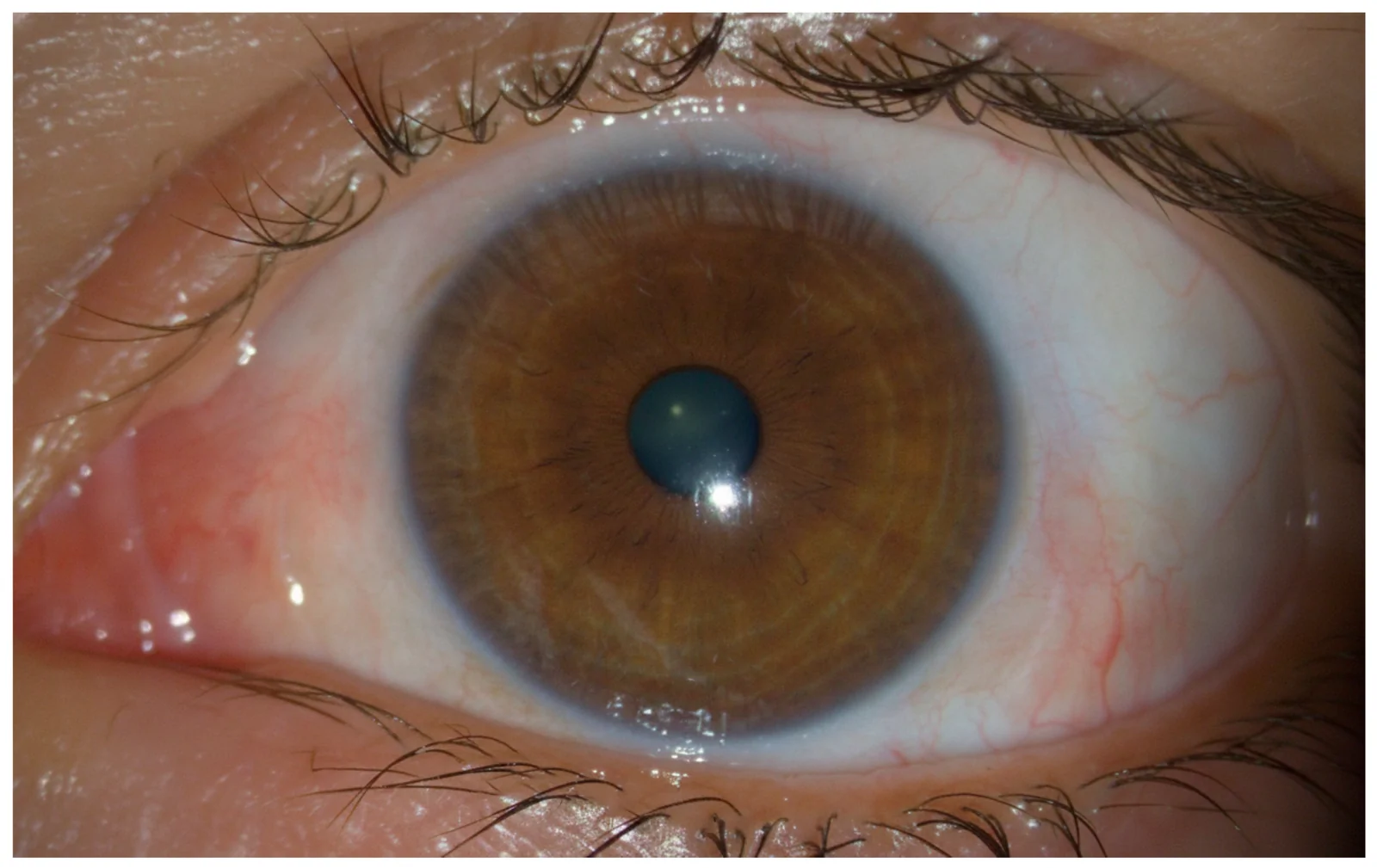
Slit-lamp photograph of the left eye after three months of ibrutinib therapy, showing marked regression of the conjunctival nodule with only mild residual hyperaemia.

Treatment options were reviewed in a joint haematology–oncology and ophthalmology meeting and included observation, repeat surgical excision, local external-beam radiotherapy and systemic targeted therapy. The lesions had already been excised on two occasions and had recurred as symptomatic nodules, indicating that local excision alone had not provided durable control. Because CLL/SLL is a systemic lymphoproliferative disorder, systemic therapy was preferred over a further local approach, and, in keeping with contemporary frameworks that endorse covalent Bruton tyrosine kinase inhibitors as a preferred first-line option [8], ibrutinib 420 mg once daily was started, together with antimicrobial prophylaxis with acyclovir and cotrimoxazole. By three months, the nodules had markedly regressed on slit-lamp examination, with only mild residual hyperaemia (Figure 3).

A complete ocular response was sustained over two years of continuous oral therapy. The response was assessed clinically at each visit by slit-lamp biomicroscopy, with a complete response being defined as disappearance of the nodular conjunctival infiltrate with no more than residual hyperaemia; no repeat conjunctival biopsy or ocular imaging was performed, and no radiological response assessment was applicable in the absence of measurable extra-ocular disease. Ophthalmological examination was carried out in parallel with haematological review every three to six months, which showed no lymphocytosis, cytopenia, lymphadenopathy, constitutional symptoms or other evidence of systemic progression.

## 3. Discussion

This case highlights the diagnostic challenge of conjunctival CLL/SLL presenting as persistent inflammatory-appearing conjunctival disease, which was managed as such for approximately one year before the diagnosis was established.

Several features sustained that interpretation. The multilobulated hyperaemic nodules were indistinguishable from nodular episcleritis, an overlap well recognised for conjunctival lymphoma [1]. Early symptomatic improvement on topical anti-inflammatory treatment reinforced the impression, although such a response reflects the suppression of secondary inflammation rather than of the underlying disease; what carried diagnostic weight was the failure of the nodules themselves to resolve. The elevated serum angiotensin-converting enzyme and lysozyme were then taken to support sarcoidosis. Once the lymphoma was diagnosed and with thoracic computed tomography, bronchoscopy and bronchoalveolar lavage having shown no granulomatous disease, both were reinterpreted as non-specific: neither enzyme is specific for sarcoidosis, no further evidence of granulomatous disease emerged during follow-up, and no causal relationship with the lymphoma can be established. The cumulative effect was diagnostic anchoring.

Ophthalmic involvement by CLL is uncommon: a systematic review identified only 123 published cases, in which conjunctival disease was among the least frequent presentations [3]. The published cases fall into two groups: secondary conjunctival infiltration in patients with an established haematological diagnosis, managed for months as chronic conjunctivitis or another inflammatory disorder [9,10]; and primary conjunctival SLL without known systemic disease, of which the few reported examples have been unilateral, with negative or indeterminate staging [4,5]. Our patient belongs to neither group, in that the disease was primary yet bilateral and multifocal, a distribution previously described essentially only in patients already known to have systemic CLL. To our knowledge this presentation has not been reported before, although the assertion rests on a non-systematic literature search, and such cases are almost certainly under-reported. The recurring theme is not the identity of the mimic, but that, in each report, a lesion that behaved as though inflammatory yet failed to resolve proved to be lymphoma.

The difficulty was compounded at the histological level. Low-grade B-cell lymphomas, in particular extranodal marginal-zone lymphoma and SLL, can closely resemble reactive lymphoid hyperplasia on conventional haematoxylin and eosin staining, both showing a lymphoid infiltrate without overt cytological atypia [1], and the reactive-appearing follicle in the first specimen reinforced that reading. The decisive variable was not what the pathologist saw but what was asked: the specimen was submitted with a clinical question about granulomatous disease, and within that framing there was no indication to apply CD5, CD23, cyclin D1 or CD10, to assess κ and λ light-chain restriction, or to request molecular clonality. The request form, rather than the tissue block, determined the delay. The first specimen was not re-examined with a lymphoma-directed panel once the diagnosis had been established; so, it cannot be stated with certainty that the neoplastic population was already present at that time, although recurrence of an identical lesion at the excised site makes this likely. Persistence and progression to bilateral multifocal disease should lower the threshold for repeat biopsy with directed immunophenotyping; when that panel was applied, the CD20-positive, CD5-positive and CD23-positive profile, with negative cyclin D1 and CD10, was diagnostic of CLL/SLL [1,7].

Molecular assessment of immunoglobulin heavy-chain (IGH) gene rearrangement, usually by multiplex PCR with standardised primer sets [11], was not performed in this patient. At the first biopsy it was not requested, for the same reason the immunohistochemical panel was not: the working diagnosis was inflammatory. At the second, it was not required, because a CD20-positive B-cell population co-expressing CD5 and CD23 with negative cyclin D1 and CD10 already satisfies the accepted immunophenotypic criteria for CLL/SLL [7]. Its value would have been retrospective: applied to the first specimen, a clonal IGH rearrangement in a lesion reported as reactive lymphoid hyperplasia might have shortened a delay of approximately one year, bearing in mind that clonality is not synonymous with malignancy and must be interpreted alongside morphology and immunophenotype [12].

The differential diagnosis includes reactive lymphoid hyperplasia, IgG4-related ophthalmic disease [13], extranodal marginal-zone (MALT) lymphoma, mantle cell lymphoma and follicular lymphoma. Distinction relies primarily on immunophenotypic and serological findings rather than on clinical appearance (Table 1). Here, CD5 and CD23 co-expression with absent cyclin D1 excluded mantle cell and marginal-zone lymphoma, and the absence of CD10 excluded follicular lymphoma. None of these distinctions can be made at the slit lamp: the entire differential collapses onto the panel applied to the biopsy.

The choice of systemic ibrutinib over local therapy was the most debated decision in this case, and three considerations converged. First, CLL and SLL are the same entity, distinguished only by the presence or absence of a leukaemic phase [7]; unlike extranodal marginal-zone lymphoma, which can be a genuinely localised mucosal neoplasm curable by local treatment, CLL/SLL is by definition systemic, and negative staging establishes that the disease burden outside the conjunctiva lies below the threshold of detection, not that the clone is confined to it. Second, local control had already failed: the lesions had in effect been excised twice and recurred on both occasions as symptomatic nodules, and excision alone is not reliably curative in ocular adnexal lymphoma [1,2]. Low-dose external-beam radiotherapy is highly effective in indolent ocular adnexal lymphoma [1], but bilateral multifocal disease would have required irradiating both ocular surfaces, with the cumulative risk of ocular surface, lacrimal and lenticular toxicity in a woman in her early fifties with 20/20 acuity and decades of life expectancy. Third, the patient met a recognised indication for systemic treatment independently of staging, since the iwCLL criteria for active disease include symptomatic extranodal involvement [7], and European and North American frameworks endorse covalent Bruton tyrosine kinase inhibitors and BCL2 inhibitors as preferred first-line options [8,14,15]. This reasoning is not a recommendation: the evidence in isolated extranodal CLL/SLL is limited and largely extrapolated from nodal and leukaemic disease, and observation would have been defensible had the disease been asymptomatic.

Long-term surveillance is the principal ongoing clinical obligation here, and two years of follow-up should be read as an early observation rather than as an outcome. Conjunctival lymphoid tumours that appear localised at presentation progress to systemic lymphoma in 7%, 15% and 28% of patients at one, five and ten years respectively, with a higher risk in bilateral disease [6]; so, our patient sits in the higher-risk group over an interval extending well beyond the period observed. The indeterminate cervical nodes and osteomedullary hypermetabolism were not characterised further and, although clinically silent, cannot be discounted, and ophthalmic involvement by CLL is itself readily under-recognised [3]. CLL/SLL also carries a lifelong risk of progression and of Richter transformation, and ibrutinib requires continuing monitoring for atrial fibrillation, hypertension, bleeding and infection for as long as it is administered [8,15]; so, haematological review at three-to-six-monthly intervals with parallel ophthalmological examination should continue indefinitely rather than to a defined endpoint. The remaining limitations are those inherent to a single case: no inference can be drawn about the optimal management of isolated conjunctival CLL/SLL, the follow-up remains short for an indolent lymphoma, and the diagnostic specimen was not further assessed by CD38 immunostaining or molecular clonality analysis. In practice, a conjunctival nodule that persists or recurs despite anti-inflammatory treatment should be regarded as potentially neoplastic and sampled with a directed immunophenotypic panel rather than managed empirically.

## 4. Conclusions

Conjunctival small lymphocytic lymphoma is rare and, in its indolent nodular form, can closely resemble inflammatory ocular surface disease. In this patient, it was treated as episcleritis and investigated for sarcoidosis for approximately one year before the diagnosis was established. The single most informative sign in retrospect was the dissociation between the symptomatic relief on topical corticosteroid and the failure of the nodules themselves to resolve. A presumed inflammatory conjunctival nodule that fails to regress or recurs, particularly when bilateral, should therefore prompt a repeat biopsy with directed immunophenotyping and consideration of molecular clonality assessment, because routine histology does not reliably distinguish low-grade lymphoma from reactive lymphoid hyperplasia. The distinction from the more common marginal-zone lymphoma is clinically important, because CLL/SLL is a systemic neoplasm that requires systemic treatment when symptomatic and long-term surveillance even when initial staging is negative. Once correctly identified, the disease responded rapidly and durably to ibrutinib, but a complete ocular response does not close the case: given the indolent biology and the higher risk conferred by bilateral disease, surveillance for delayed systemic progression must continue indefinitely.

## Figures and Tables

**Table 1:** Distinguishing conjunctival CLL/SLL from its principal mimics.

Entity	Key Morphology	CD5	CD23	Cyclin D1	Decisive Feature
Reactive lymphoid hyperplasia	Polymorphous infiltrate; well-formed germinal centres with polarised mantle zones	–	– (on B cells)	–	Polytypic κ/λ; no clonal IGH rearrangement
IgG4-related ophthalmic disease	Dense lymphoplasmacytic infiltrate, often storiform fibrosis; lacrimal gland, frequently bilateral	–	–	–	IgG4+/IgG+ ≥ 40% or >50 IgG4+ cells/HPF; serum IgG4 > 135 mg/dL
Extranodal marginal-zone (MALT) lymphoma	Monocytoid/centrocyte-like cells, plasmacytic differentiation, lymphoepithelial lesions	–	–	–	≈80% of ocular adnexal lymphoma; light-chain restricted, CD5–/CD23–
Mantle cell lymphoma	Monotonous small-to-medium cells with irregular nuclei	+	–	+	Cyclin D1 positivity with CD23 negativity
Follicular lymphoma	Follicular growth pattern, centrocytes and centroblasts	–	–	–	CD10+, BCL6+, BCL2+
CLL/SLL (this case)	Diffuse infiltrate of small mature lymphocytes, condensed chromatin, no germinal centres	+	+	–	CD5/CD23 co-expression with cyclin D1 and CD10 negativity

CLL/SLL, chronic lymphocytic leukaemia/small lymphocytic lymphoma; HPF, high-power field; IGH, immunoglobulin heavy chain; MALT, mucosa-associated lymphoid tissue.

## Data Availability

The original contributions presented in this study are included in the article. Further inquiries can be directed to the corresponding author.
